# Patient Engagement in a Multimodal Digital Phenotyping Study of Opioid Use Disorder

**DOI:** 10.2196/45556

**Published:** 2023-06-13

**Authors:** Cynthia I Campbell, Ching-Hua Chen, Sara R Adams, Asma Asyyed, Ninad R Athale, Monique B Does, Saeed Hassanpour, Emily Hichborn, Melanie Jackson-Morris, Nicholas C Jacobson, Heather K Jones, David Kotz, Chantal A Lambert-Harris, Zhiguo Li, Bethany McLeman, Varun Mishra, Catherine Stanger, Geetha Subramaniam, Weiyi Wu, Christopher Zegers, Lisa A Marsch

**Affiliations:** 1 Division of Research Kaiser Permanente Northern California Oakland, CA United States; 2 Department of Psychiatry and Behavioral Sciences University of California San Francisco San Francisco, CA United States; 3 Kaiser Permanente Bernard J Tyson School of Medicine Pasadena, CA United States; 4 Center for Computational Health IBM Research Yorktown Heights, NY United States; 5 Addiction Medicine and Recovery Services The Permanente Medical Group Northern California Oakland, CA United States; 6 Addiction Medicine and Recovery Services The Permanente Medical Group Northern California Vallejo, CA United States; 7 Center for Technology and Behavioral Health Geisel School of Medicine Dartmouth College Lebanon, NH United States; 8 Department of Biomedical Data Science Geisel School of Medicine Dartmouth College Lebanon, NH United States; 9 Department of Computer Science Dartmouth College Hanover, NH United States; 10 Profit Intelligence Amazon.com Seattle, WA United States; 11 Khoury College of Computer Sciences Northeastern University Boston, MA United States; 12 Department of Health Sciences Northeastern University Boston, MA United States; 13 Center for Clinical Trials Network National Institute on Drug Abuse Bethesda, MD United States; 14 Addiction Medicine and Recovery Services The Permanente Medical Group Northern California Sacramento, CA United States

**Keywords:** opioid use disorder, digital phenotyping, medication for opioid use disorder, MOUD, ecological momentary assessment, EMA, passive sensing, social media, opioid, OUD, data collection, smartphone, digital health

## Abstract

**Background:**

Multiple digital data sources can capture moment-to-moment information to advance a robust understanding of opioid use disorder (OUD) behavior, ultimately creating a digital phenotype for each patient. This information can lead to individualized interventions to improve treatment for OUD.

**Objective:**

The aim is to examine patient engagement with multiple digital phenotyping methods among patients receiving buprenorphine medication for OUD.

**Methods:**

The study enrolled 65 patients receiving buprenorphine for OUD between June 2020 and January 2021 from 4 addiction medicine programs in an integrated health care delivery system in Northern California. Ecological momentary assessment (EMA), sensor data, and social media data were collected by smartphone, smartwatch, and social media platforms over a 12-week period. Primary engagement outcomes were meeting measures of minimum phone carry (≥8 hours per day) and watch wear (≥18 hours per day) criteria, EMA response rates, social media consent rate, and data sparsity. Descriptive analyses, bivariate, and trend tests were performed.

**Results:**

The participants’ average age was 37 years, 47% of them were female, and 71% of them were White. On average, participants met phone carrying criteria on 94% of study days, met watch wearing criteria on 74% of days, and wore the watch to sleep on 77% of days. The mean EMA response rate was 70%, declining from 83% to 56% from week 1 to week 12. Among participants with social media accounts, 88% of them consented to providing data; of them, 55% of Facebook, 54% of Instagram, and 57% of Twitter participants provided data. The amount of social media data available varied widely across participants. No differences by age, sex, race, or ethnicity were observed for any outcomes.

**Conclusions:**

To our knowledge, this is the first study to capture these 3 digital data sources in this clinical population. Our findings demonstrate that patients receiving buprenorphine treatment for OUD had generally high engagement with multiple digital phenotyping data sources, but this was more limited for the social media data.

**International Registered Report Identifier (IRRID):**

RR2-10.3389/fpsyt.2022.871916

## Introduction

### Buprenorphine Treatment for Opioid Use Disorder

The opioid crisis continues unabated in the United States, with 2.7 million individuals diagnosed with past-year opioid use disorder (OUD) in 2020 [1] and over 100,000 fatal overdoses from opioids in the 12 months ending in May 2022 [2]. Buprenorphine is an effective medication for treating OUD, reducing opioid use, and lowering mortality risk; yet, many do not remain in treatment, and the risk of relapse is high [3-6]. Understanding the risk of relapse and treatment discontinuation is critical to addressing the opioid crisis.

### Digital Phenotyping

Digital phenotyping is the use of personal digital technologies to collect and analyze moment-to-moment data in the study of human behavior and function [7,8]. Such technologies enable high-frequency data collection, either continuously or multiple times per day, which makes them well-suited for capturing the episodic nature of opioid use as well as the dynamic environmental (eg, social situations) and internal factors (eg, stress) that may predict its use [9]. Relative to conventional data collection methods, digital phenotyping methods can capture transient events for a richer understanding of OUD.

Common digital phenotyping methods include ecological momentary assessment (EMA), wearable devices that generate passive sensing data, and, increasingly, social media data. EMA involves individuals actively responding to frequent prompts on a mobile device (eg, a smartphone) about their behavioral, cognitive, affective, or functional states throughout the day [9,10]. The respondent burden can be a concern with EMA [11], although EMA studies among patients with substance use disorders, including OUD, have shown generally acceptable compliance rates, although with considerable variation [11,12]. Sensing data from wearable devices such as smartwatches, which continuously measure passive physiological and behavioral data (eg, heart rate and sleep), have a lower participant burden and a lower likelihood of missing data than self-report [13,14].

### Prior Work on Engagement in EMA, Wearable Sensors, and Social Media Data

Much of the research using EMA data among patients with OUD has been conducted by researchers at the National Institute on Drug Abuse’s intramural research center [15,16], studying how internal states and health behaviors (eg, stress and craving) are related to opioid use. These studies have demonstrated considerable success using these digital methodologies; however, the setting was a treatment-research clinic, where patient compliance may be higher than that in community-based clinic settings. There are a few studies that have used wearable sensor data among patients treated for OUD [10,17,18].

Social media data can also provide important insights into a person’s behavioral health status through both content and usage [19,20]. Social media data have been shown to predict alcohol use and problems, as well as depression [21-23], and have been used for surveillance of opioid problems [24]. To our knowledge, this method has not been studied among clinical samples of individual patients with OUD to predict opioid use.

### Study Aims

Digital phenotyping studies of patients with OUD have typically used a single digital method, but the prediction of relapse events and treatment discontinuation may be improved with the combined use of digital methods, and some digital data sources may more strongly predict different clinical trajectories and outcomes than others. Patient engagement in a study involving multiple digital data streams has not previously been studied in this important patient population [9,25,26]. The overall goal of the study, “Harnessing Digital Health Technologies to Understand Clinical Trajectories of Opioid Use Disorder” (D-TECT; CTN-0084-A2), was to determine patient engagement with simultaneous collection of EMA, passive sensing, and social media data among patients receiving buprenorphine for OUD. Findings can inform how future studies can use these methods in combination to better understand and predict opioid use behaviors and ultimately inform personalized interventions to improve buprenorphine treatment for OUD.

## Methods

### Study Design

The detailed design and protocol of the D-TECT study have been presented elsewhere [27]. Briefly, this observational study collected multiple streams of digital data over a 12-week study period—EMA data from a smartphone app, passive sensor data through a smartphone and a smartwatch, and social media data from participants’ social media accounts. Questionnaires, electronic health record (EHR) data, and claims data were also collected.

### Participants and Setting

Participants were recruited from four Kaiser Permanente Northern California (KPNC) Addiction Medicine Recovery Services programs between June 2020 and January 2021. KPNC is a large, integrated health care delivery system serving approximately 4.5 million members and providing care through individual, commercial, Medicare, and Medicaid plans. Members are racially and socioeconomically diverse (9% Black, 22% Asian/Pacific Islander, 18% Latino, 50% non-Hispanic White; 17% <200% above the federal poverty level, 20% with some post–high school education, 54% with a 4-year college degree or above) and generally representative of the region’s population with access to care [28].

Buprenorphine is available to patients with OUD along with group-based treatment modalities that involve a combination of supportive group therapy, education, cognitive behavioral therapy, family-oriented therapy, and individual treatment. Individual counseling and physician appointments are available as needed. Intensive treatment is required for up to 9 weeks, with reduced intensity and maintenance phases up to 1-year post intake. During the COVID-19 pandemic, treatment was almost, exclusively, virtual.

### Eligibility and Recruitment

Participants had to meet several eligibility criteria: age 18 years or older, treatment with buprenorphine for OUD for 2 weeks prior to initial study contact, KPNC membership at the time of consent, ability to understand English, and permission to access EHR and claims data. Participants had to be able to participate in the study for 12 weeks, be willing to carry and use a smartphone (study provided or their own) and be willing to wear a study-provided smartwatch continuously for 12 weeks. Participants were identified and screened through EHR data, including a review of medical records. After eligibility was established, an informational letter was sent to the individual’s email address through the web-based KPNC patient portal. A research associate followed up within 1 week to further explain the study, screen for eligibility, and obtain consent. Given the COVID-19 pandemic, all contacts were virtual.

### Ethics Approval

The study was reviewed and approved (1531090) by the KPNC Institutional Review Board in accordance with all applicable regulations. Participants provided consent after the nature and possible risks and benefits of the study were explained, which was documented with a digital signature. Participants consented to the collection of all primary and secondary data (eg, EMA, questionnaire, sensor, optional social media, and EHR) and provided Health Insurance Portability and Accountability Act authorization for the use and disclosure of protected health information for research. The informed consent form included language permitting future secondary analyses without additional consent. A consent quiz was administered to assure participants fully understood the types and scope of data being collected and that no study staff were monitoring participant data in real time [29]. All data were stored behind secure firewalls on encrypted servers, with access limited to research staff. Data were deidentified before conducting analyses.

### Procedures and Data Sources

#### Overview

Study procedures included 1-2 baseline appointments, a follow-up appointment at 12 weeks, and weekly and ad hoc check-ins as needed. All appointments were conducted by telephone. Urine tests were collected virtually at baseline and follow-up, with kits mailed to the participants and results uploaded using a secure REDCap (Vanderbilt University) electronic data capture tool hosted at Dartmouth College. Interviewers administered baseline and follow-up questionnaires to participants by telephone; each took approximately 1-1.5 hours.

#### EMA Surveys

Participants were provided with an Android phone, although 4 participants used their personal phones. Participants were asked to keep the study phone on and with them for at least 8 hours per day. The research team developed a custom EMA app that was preinstalled on study phones or downloaded by participants onto personal phones at the baseline appointment. EMA prompts were sent randomly 3 times per day (morning, afternoon, and evening). Participants could pause prompts for up to 2 hours per day at their discretion. EMAs were delivered every day of the 12-week study period. The survey was live for 1 hour after the prompt, and participants had 15 minutes to complete it.

EMA surveys included 20 questions on sleep, stress, pain severity, pain interference, pain catastrophizing, craving, withdrawal, substance use risk context, mood, context, substance use, self-regulation, buprenorphine adherence, and the impact of COVID-19. Detailed descriptions of survey measures are provided elsewhere [27]. Participants were asked to self-initiate EMA responses if cocaine or nonprescribed opioid use occurred anytime during the 12-week study period.

Research staff monitored the speed of participants’ responses and reached out to those whose fast response times suggested a lack of attention or engagement to encourage them to take their time. No participants were excluded for their fast response times.

#### Smartphone Passive Data

App usage, audio or conversation, call or text, GPS, screen on or off, phone lock or unlock, phone notification information, Wi-Fi and Bluetooth logs, and physical activity state (eg, still, walking, or in a vehicle) were collected via the smartphone.

#### Smartwatch Sensor Data

Participants were provided with a Garmin Vivosmart 4 smartwatch and asked to wear it continuously (except during predetermined exception periods, such as when showering or charging the device) for a minimum of 18 hours per day. The wearable data were synced directly with Garmin (we did not have direct access to raw sensor data). We used the Garmin Health Connect API [30] to obtain various health metrics computed by Garmin’s proprietary algorithm, such as heart rate, sleep stage information, stress levels, physical activity levels, and step counts.

#### Social Media Data

Participants were asked to provide data from their Facebook, Instagram, and Twitter accounts—all historical postings since they established their accounts plus activity during the 12-week study period. Social media posts were counted differently based on platform: Facebook posts were text and image posts, Instagram posts were images only, and Twitter posts consisted only of tweets (text posts). Live stories and videos were not counted as posts on any platform. Participants could decline to provide their social media data and still participate in the rest of the study. Social media data were downloaded twice—at baseline (start of week 1) and again at follow-up (end of week 12). The first download ensured all historical posts were captured, even if the participant left the study or did not complete the second download. Participants requested access to their data from the social media platforms and directly downloaded posts from the platforms to a secure study server using a remote desktop procedure. Participants were told that the research staff did not monitor social media posts and that they should not change their social media behavior during the study.

### Compliance and Participant Tracking

We developed a dashboard to track EMA responses, phone “on” status, watch wear time, and participant reimbursement. Payments were automatically distributed weekly via reloadable debit cards. If participants were not compliant or participating as expected, study staff would contact them to troubleshoot. Regardless of dashboard status, the study team communicated weekly with participants by phone or text message to encourage participation and provide incentive updates.

### Reimbursement

Participants were compensated for each piece of digital and self-reported data, for a total possible compensation of US $995. Participants could receive up to US $21 per week for completing EMAs; a $10 per week bonus if they completed a minimum number of EMAs; and up to US $14 per week for carrying the smartphone and wearing the smartwatch. In addition, participants who completed at least 80% of EMA surveys in a given week were entered into a weekly drawing for US $50. Participants who provided social media data could receive an additional US $180 if they provided data from all 3 platforms at baseline and follow-up. Reimbursement for digital data collection occurred through a reloadable debit card. Participants also received US $75 and US $100 Target gift cards for completing the baseline and follow-up questionnaires, respectively, and up to US $100 on the reloadable debit card for returning the study smartphone and smartwatch.

### Statistical Analyses

The primary outcomes included the following: (1) phone carrying is the percentage of days during the 12-week study period during which participants carried their smartphone for ≥8 hours per day, calculated as the number of days the criteria were met divided by 83 study days (day 1 was omitted to account for different start times); (2) watch wearing is the percentage of days during the 12-week period participants wore the smartwatch for ≥18 hours per day, calculated as the number of days the criteria were met divided by 83 study days; (3) response rate (RR) to EMA prompts during the 12-week phase; (4) the percentage of participants who consented to social media data download among those who had accounts; and (5) sparsity of social media data as measured by the number of posts per “downloader” (person who downloaded their posts) at baseline and follow-up, and the number of new posts gained at the follow-up download compared to baseline. Secondary measures include the mean number of hours during which the phone was carried, the watch was worn, and the hours of sleep per day. If the phone and watch were on and data were collected and transmitted, the criteria for phone carrying and watch wearing were satisfied.

We generated descriptive statistical summaries of phone carrying and watch wearing metrics, the mean EMA RR for the overall study period, and the sparsity of social media data. We also provided a visualization of the mean and median EMA RR in each study week and tested for the presence of a monotonic trend in the mean and median over the study period using the nonparametric Mann-Kendall test. We performed statistical inference to compare statistics (ie, means) across age, gender, and racial or ethnic subgroups. When reporting outcome measures computed over the entire study period, we excluded data from the first day of the study since participants may not have been active for the entire day.

## Results

### Participant Flow and Characteristics

**Figure 1:**
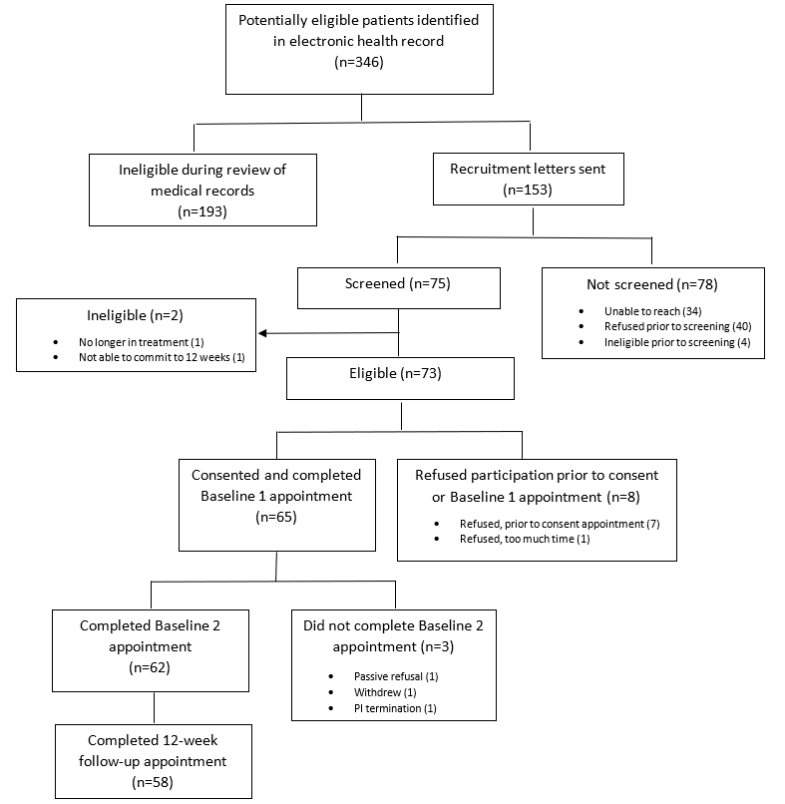
Flow diagram for screening, enrollment, and participation.

Between June 2020 and January 2021, a total of 346 individuals were identified in the EHR as potentially meeting study criteria, and 73 were determined eligible after EHR review and recruitment calls. Of them, 65 individuals consented, for an overall recruitment rate of 89% (Figure 1).

The average age of the sample was 37 years (Table 1). The sample was 47% female, 71% White, and 21% Hispanic; 63% of participants had an education beyond high school, 68% of them were currently employed, and 44% of them were married or living with a partner. The mean household income was US $65,089. At baseline, approximately 13% of participants had major depressive disorder in the past month, 11% of them had generalized anxiety disorder (in the past 6 months), and 11% of them had ADHD (in the past 6 months). At baseline, 77% of participants had an OUD diagnosis in the past 12 months (although receiving buprenorphine for OUD was an inclusion criterion); other baseline substance use disorders in the past 12 months included alcohol (36%), sedatives (32%), cannabis (31%), and stimulants or cocaine (23%).

**Table 1:** Characteristics of consented participants (N=62).

Characteristics			Values
**Gender, n (%)^a^**			
	Male	31 (50)	
	Female	29 (47)	
Age (years), mean (SD)			37.2 (13.3)
**Ethnicity, n (%)**			
	Hispanic, Latino, or of Spanish origin	13 (21)	
	Non-Hispanic	49 (79)	
**Race, n (%)^b^**			
	White	44 (71)	
	Non-White or more than one race	18 (29)	
**Household income (US $), n (%)**			
	0-25,000	15 (24)	
	25,001-50,000	13 (21)	
	50,001-75,000	16 (26)	
	75,001-100,000	10 (16)	
	>100,000	8 (13)	
Household income (US $), mean (SD)			65,089 (57,318)
**Education, n (%)**			
	High school graduate, GED^c^, or less	23 (37)	
	Some college or no degree	20 (32)	
	Associate’s degree	14 (23)	
	College graduate or postgraduate	5 (8)	
**Employment status, n (%)**			
	Employed	42 (68)	
	Unemployed	12 (19)	
	Student, retired, or disabled	8 (13)	
**Marital status, n (%)**			
	Married or living with partner	27 (44)	
	Never married	25 (40)	
	Divorced, separated, or widowed	10 (16)	
**Mental health diagnosis, n (%)^d,e^**			
	Major depressive episode in the past month	8 (13)	
	Generalized anxiety disorder in the past 6 months	7 (11)	
	Adult attention deficit hyperactivity disorder in the past 6 months	7 (11)	
	Other diagnoses	9 (14)	
**Substance use disorder (past 12 months), n (%)^d,f^**			
	Alcohol use disorder	22 (36)	
	Cannabis use disorder	19 (31)	
	Stimulant or cocaine use disorder	14 (23)	
	Opioid use disorder	48 (77)	
	Sedative, hypnotic, or anxiolytic disorder	20 (32)	
	Other use disorders	4 (6)	

^a^Due to small cell sizes, we do not report gender identities other than male and female.

^b^Non-White includes Asian, Black, and Other, and were collapsed because of small cell sizes. There were no Native American, Pacific Islander, American Indian, or Alaska Native participants.

^c^GED: General Educational Development.

^d^Diagnoses based on diagnostic instrument, NetSCID-V.

^e^Other was collapsed due to small cell sizes and includes social anxiety disorder (past 6 months), depressive disorder due to another medical condition (past month), depressive disorder induced by substance use (past month), panic disorder (past month), and posttraumatic stress disorder (past month). There were no diagnoses of a manic episode.

^f^Other includes hallucinogen use disorder and inhalant use disorder. There were no diagnoses of phencyclidine use disorder.

Participants who dropped out, withdrew, or were terminated after consent but prior to receiving or activating the study phone or watch were not included in the analysis (n=3) because they were not able to contribute any data during the active study phase or at follow-up. Thus, the following analyses include 62 participants.

### Primary Outcomes

#### Percentage of Study Days on Which Participants Met Criteria

Most participants had high compliance with study criteria each week of the study period (Multimedia Appendices 1-3), so we present the overall means for the study period. On average, participants met phone carrying criteria on 94% of study days, met watch wearing criteria on 74% of study days, and wore the watch to sleep on 77% of study days (Table 2). We found no significant differences by gender, age, race, or ethnicity for any of the 3 metrics. The mean phone carrying time of 21 (SD 3) hours per day and mean watch wear time of 19 (SD 4) hours per day exceeded minimum study requirements, and mean sleep hours were 5 (SD 2) (Multimedia Appendices 4-6).

**Table 2:** Percentage of study days on which participants met criteria for digital technology (N=62).

		Percentage of days on which participants carried their phone for ≥8 hours/day		Percentage of days on which participants wore their watch for ≥18 hours/day			Percentage of days on which participants wore their watch while sleeping	
		Mean (SD)	95% CI	Mean (SD)	95% CI	Mean (SD)		95% CI
All participants		94.2 (13.3)	(90.8-97.6)	73.7 (13.3)	(67.7-79.7)	77.1 (21.4)		(71.7-82.5)
**Gender identity^a^**								
	Female (n=29)	93.7 (11.6)	(89.3-98.1)	75.2 (11.6)	(67.5-82.8)	79.0 (19.8)		(71.5-86.5)
	Male (n=31)	94.2 (15.3)	(88.6-99.9)	72.5 (15.3)	(62.6-82.5)	75.3 (23.6)		(66.7-84.0)
**Age group (years)**								
	18-29 (n=21)	90.4 (19.6)	(81.4-99.3)	70.0 (19.6)	(58.7-81.3)	73.1 (23.2)		(62.5-83.6)
	30-49 (n=30)	95.6 (9.2)	(92.1-99.0)	74.5 (9.2)	(66.5-82.5)	78.6 (20.1)		(71.1-86.2)
	≥50 (n=11)	97.6 (4.7)	(94.4-100.8)	78.6 (4.7)	(60.5-96.8)	80.5 (22.0)		(65.7-95.3)
**Ethnicity**								
	Hispanic, Latino or of Spanish origin (n=13)	97.1 (5.0)	(94.1-100.1)	78.1 (5.0)	(64.2-92.1)	78.9 (20.7)		(66.3-91.4)
	Not Hispanic, Latino or of Spanish origin (n=49)	93.4 (14.7)	(89.2-97.6)	72.5 (14.7)	(65.7-79.3)	76.6 (21.7)		(70.4-82.9)
**Race**								
	White (n=44)	93.9 (14.5)	(89.5-98.3)	76.1 (14.5)	(69.2-83.0)	79.1 (20.8)		(72.8-85.5)
	Non-White or >1 race (n=18)	94.8 (10.2)	(89.8-99.9)	67.8 (10.2)	(55.4-80.2)	72.1 (22.6)		(60.9-83.3)

**^a^**Summary statistics are not reported for participants who reported their gender identity as “Nonbinary” or “Prefer not to report” because there were fewer than 5 participants who selected either of these categories.

#### EMA Response Rates

**Figure 2:**
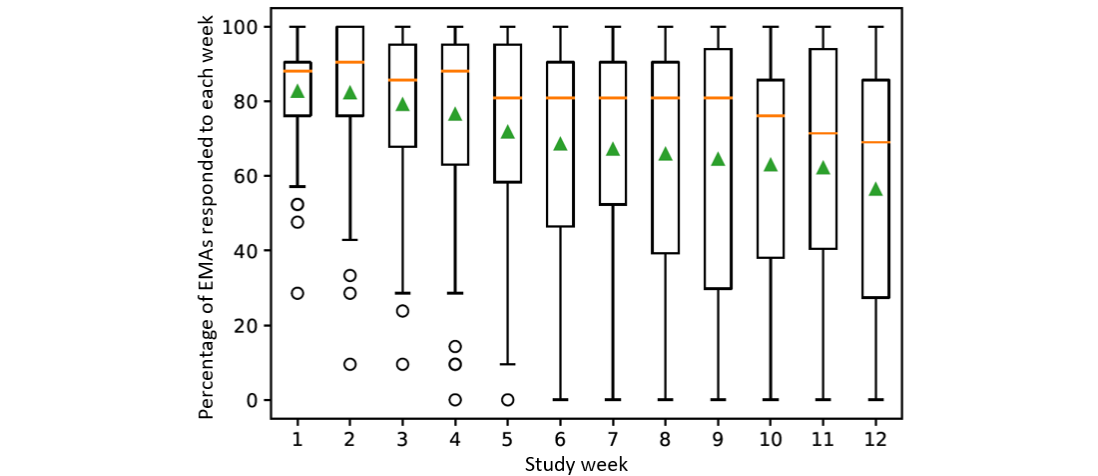
Ecological momentary assessment (EMA) response rate by week. Orange line indicates median and green triangle indicates mean. Both have a significant, monotonically decreasing trend.

Figure 2 shows the average percent of EMA prompts responded to out of the prompts sent by week. The mean EMA RR across the study period was 70% (SD 26%), declining from 83% (SD 15%) to 56% (SD 39%) from week 1 to week 12, with a significant, monotonically decreasing trend (Kendall =–0.10, *P*<.001). Median RRs also declined from 88% in week 1 to 69% in week 12, with a significant monotonically decreasing trend (Kendall =–0.77, *P*<.001) and with variability increasing in later weeks. Mean RRs were similar by the time of day (69%, SD 26% for morning, 71%, SD 25% for afternoon, and 71%, SD 27% for evening [data not shown]).

The mean EMA RRs were not significantly different by gender, ethnicity, or race (Table 3). The average time to respond to the EMA prompts was 4.0 (SD 1.3) minutes. Ten participants submitted a total of 17 self-initiated surveys.

**Table 3:** Ecological momentary assessment (EMA) response rate over study period by gender, age, ethnicity, and race (N=62).^a^

		Participants		
		n	Mean (SD)	95% CI
		62	70.0 (25.7)	(63.5-76.6)
**Gender identity**				
	Female	29	66.5 (27.6)	(56.0-77.0)
	Male	31	72.5 (24.4)	(63.5-81.4)
**Age group (years)**				
	18-29	21	59.4 (26.5)	(47.4-71.5)
	30-49	30	73.9 (25.6)	(64.3-83.5)
	50 years and older	11	79.7 (18.1)	(67.6-91.9)
**Ethnicity**				
	Hispanic, Latino, or of Spanish origin	13	70.6 (27.8)	(53.8-87.4)
	Not Hispanic, Latino, or of Spanish origin	49	69.9 (25.4)	(62.6-77.2)
**Race**				
	White	44	72.0 (25.3)	(64.3-79.7)
	Non-White or >1 race	18	65.2 (26.6)	(52.0-78.4)

**^a^**Denominator is the number of study days minus the first day (n=83).

#### Social Media Data Consent and Download

Approximately 90% of participants reported having one of the three social media accounts; Facebook was the most common (84%). Only half of the participants had a Twitter account. Of the participants who owned social media accounts, a subset consented to provide their social media data. Consent rates among those who had any accounts were high (88% among all platforms) and ranged from 89%-94% per platform (Table 4). There were no significant differences in consent rates by participant characteristics.

Fewer participants downloaded their data than provided consent (Table 5). Among those who consented to provide social media data, 55% of Facebook, 54% of Instagram, and 57% of Twitter participants actually downloaded their data at baseline or follow-up. There were no significant differences in these rates by gender, age, race, or ethnicity.

**Table 4:** Percentage and number of participants consenting to download Facebook, Instagram, and Twitter posts, among those with accounts.

		Facebook, % (n/n)	Instagram, % (n/n)	Twitter, % (n/n)	Any, % (n/n)
Participants with accounts		88 (46/52)	94 (43/46)	90 (28/31)	88 (49/56)
**Gender identity^a^**					
	Female	100 (27/27)	100 (22/22)	94 (15/16)	100 (27/27)
	Male	74 (17/23)	86 (19/22)	85 (11/13)	74 (20/27)
**Age group (years)**					
	18-29	94 (17/18)	90 (18/20)	86 (12/14)	90 (19/21)
	30-49	96 (23/24)	95 (20/21)	93 (13/14)	96 (24/25)
	≥50	60 (6/10)	100 (5/5)	—^b^	60 (6/10)
**Ethnicity**					
	Hispanic, Latino, or Spanish origin	100 (13/13)	100 (12/12)	100 (6/6)	100 (13/13)
	Not Hispanic, Latino, or Spanish origin	85 (33/39)	91 (31/34)	88 (22/25)	84 (36/43)
**Race**					
	White	84 (31/37)	94 (29/31)	91 (20/22)	85 (34/40)
	Non-White or >1 race	100 (15/15)	93 (14/15)	89 (8/9)	94 (15/16)

^a^Values are not reported for participants who reported their gender identity as “Nonbinary” or “Prefer not to report” because there were fewer than 5 participants who selected either of these categories.

^b^Values not reported due to small cell sizes.

**Table 5:** Percent and number of participants who downloaded social media data at baseline or follow-up, among those who consented to download.

		Facebook, % (n/n)	Instagram, % (n/n)	Twitter, % (n/n)
Participants who consented to download		54 (25/46)	54 (23/43)	57 (16/28)
**Gender identity^a^**				
	Female	56 (15/27)	55 (12/22)	53 (8/15)
	Male	47 (8/17)	47 (9/19)	55 (6/11)
**Age group (years)**				
	18-29	59 (10/17)	67 (12/18)	67 (8/12)
	30-49	56 (13/23)	45 (9/20)	54 (7/13)
	≥50	33 (2/6)	—^b^	—^b^
**Ethnicity**				
	Hispanic, Latino, or Spanish origin	38 (5/13)	42 (5/12)	—^b^
	Not Hispanic, Latino, or Spanish origin	60 (20/33)	58 (18/31)	55 (12/22)
**Race**				
	White	59 (18/31)	59 (17/29)	50 (10/20)
	Non-White or >1 race	47 (7/15)	43 (6/14)	75 (6/8)

^a^Values are not reported for participants who reported their gender identity as “Nonbinary” or “Prefer not to report” because there were fewer than 5 participants who selected either of these categories.

^b^Values not reported due to small cell sizes.

#### Sparsity of Social Media Data

We calculated the mean number of posts per downloader (MPPD) over the history of their accounts at baseline and at follow-up (Multimedia Appendix 7). Across all platforms, a minority of participants contributed the majority of posts. Of participants who downloaded Facebook, the historical MPPD was 1263 (SD 1603) at baseline and 1218 (SD 1532) at follow-up. Of participants who downloaded Instagram data, the historical MPPD was 213 (SD 410) at baseline and 270 (SD 464) at follow-up. Of participants who downloaded Twitter data, the historical MPPD was 316 (SD 574) at baseline and 226 (SD 582) at follow-up. No significant differences in MPPD were observed by gender, age, race, or ethnicity at baseline or follow-up. The percentage increase in new posts gained with the follow-up download, compared to the baseline, was 18% for Facebook, 17% for Instagram, and 50% for Twitter. In descriptive post hoc analyses of sparsity over time, the average weekly number of posts during the 12-week study period was 5.2 (SD 15.0) for Facebook, 0.78 (SD 1.3) for Instagram, and 0.17 (SD 0.27) for Twitter. In a comparable time frame of 12 weeks prior to baseline, the average weekly number of posts was 5.2 (SD 12.7) for Facebook, 1.23 (SD 4.0) for Instagram, and 0.08 (SD 0.16) for Twitter.

## Discussion

### Principal Results

This study examined patient engagement in collecting multiple digital phenotyping data among patients receiving buprenorphine for OUD, assessed primarily by whether the participants met study criteria for phone and watch use and EMA response. Engagement over the study period was generally high, with compliance rates consistent with the literature [11]. Importantly, engagement was high among gender, age, racial, and ethnic groups.

### Facilitators and Barriers to Engagement

The high participation rates may be due to several reasons. Participants may have found value in routinely reflecting on their clinical status and well-being (eg, in responding to EMA questions). Participants may have found the experience of providing digital data (eg, passive sensing data and EMA data) novel and easy to do. Compensation may have been effective; the incentivization of each participation component may have promoted study retention and EMA compliance. This study was carried out during the COVID-19 pandemic, when some participants may have had higher financial needs. Finally, the protocol involved the staff being in frequent touch with participants via phone calls and text when engagement decreased, as well as on an ongoing basis through a weekly encouragement SMS text message. The engagement was closely tracked through a dashboard, and engagement may have been lower without the high “touch” of the study protocol.

There was some modest waning in EMA RRs over time, as observed in other digital health studies [11,31], possibly due to diminished motivation, reduced novelty, participant burden, and external factors such as changes in work or familial obligations [11,32]. Anecdotally, some participants reported that work constraints, particularly those with shift work, made responding to randomly delivered prompts difficult because of schedule restrictions and the timing of breaks. Family obligations, particularly childcare responsibilities, were also cited as a barrier to a timely EMA response. Reducing the frequency of EMA surveys or dynamic incentive schedules (eg, escalating over time with reset contingencies) may help maintain EMA RRs over time. Participants also reported burden and fatigue with receiving the same questions over the study period, particularly for questions they felt did not apply to them (eg, withdrawal and craving). Technical issues (eg, the watch not syncing and faulty notifications) and the inconvenience of carrying a second phone were challenging for some participants. Participants may have also discontinued OUD treatment or had a relapse event, which may have reduced their motivation (although staying in treatment was not a requirement for participation).

### Engagement With Multimodal Data Sources

There are limited data on digital phenotyping with patients receiving buprenorphine for OUD who are at high risk for relapse and treatment discontinuation. Importantly, we know little about engagement in the context of collecting multiple digital data streams, particularly those that can be leveraged simultaneously for a richer understanding of relapse events, treatment outcomes, and adaptive interventions than is possible with single digital sources. In an era of heighted privacy concerns related to technology, the high enrollment is noteworthy, particularly since the staff did not have the opportunity to make personal connections or warm handoffs in the clinic due to the pandemic. While some parts of the virtual recruitment protocol were challenging (eg, setting up study phones and virtual urine drug screen), participants may have otherwise found this virtual approach to be convenient. In addition, the financial compensation likely helped with enrollment.

The use of study versus personal phones highlights potential implications for data interpretation. The study team developed a custom app that operated more smoothly on the study phone and often did not work well with personal phones. Thus, most participants had 2 phones (a study phone and a personal phone), and the extent to which participants used the study phone as they would a personal phone could have impacted the amount and representativeness of the data. On the other hand, studies that require participants to “bring their own device” may have technical challenges with compatibility, as was found in a recent study of prescription opioid and cannabis use [32]. Another data challenge was that participants’ activity levels during the pandemic may not be representative of nonpandemic times, when their daily routines may be different.

### Social Media Data Considerations

One question of considerable interest was participants’ social media participation. There was more willingness to provide these data than expected, with the vast majority of study participants consenting. However, many participants who consented to download their social media data did not follow through, which may have been due to the technical logistics involved with downloading. Originally, the study team planned to download participants’ social media data from the platforms with their consent. However, the platforms changed their policies immediately prior to the start of the study so that only participants could download their data. This requirement presented a considerable barrier for some participants; it was a complicated and tedious process that required participants to log into a remote server, request the data file from the platform, remember to check their email for a notification that their data were ready and log into a remote server to download the data to a secure study server. This multistep process was understandably too complicated for some individuals, even when carried out with a research associate on the phone with them, and likely even more challenging if participants were in an acute phase of OUD treatment. Simplified, more user-friendly methods for accessing social media data are important to increase engagement. Sparse social media data limits the interaction with the other data sources; studies interested in the combined use of these data could consider social media use as an eligibility criterion.

The amount of social media data available varied widely across participants; most had a modest number of posts, and further analyses will examine the predictive utility of the data for opioid use and medication adherence. The use of 2 opportunities to download social media posts demonstrated added value with an increased percentage of posts obtained, and research teams interested in these data could consider this approach. Finally, it is possible that participants changed their posting behavior while enrolled in the study, even after being informed that the research staff was not monitoring posts. Post hoc analyses showed higher average posting for Instagram and lower average posting for Twitter in a similar prebaseline period. However, sample sizes were small and subject to the influence of outliers, which limits the ability to draw conclusions. We note that the average number of posts for Facebook, which had the largest number of MPPDs, was stable.

### Limitations

This study was designed to examine the in-depth engagement of digital phenotyping and to apply these lessons to future studies. Although this study was promising, we note that the setting covered a population largely insured through employers or Medicare, and participants were generally young and White. This may limit generalizability, and digital literacy may be higher in this population than in other settings. The study occurred during the COVID-19 pandemic, which likely impacted participants’ substance use as well as their treatment [33]. The pandemic also significantly changed behaviors and reduced social activity with the implementation of social distancing measures [34]. Future analyses predicting outcomes will need to consider the pandemic’s social distancing impact (eg, fewer Bluetooth devices detected with fewer in-person interactions). In addition, the COVID-19 pandemic may have impacted patient engagement with the study both positively and negatively (eg, potentially more bandwidth for participating vs being overwhelmed by pandemic stressors). We do not have the participants’ COVID-19 infection status, which may have further impacted their behavior and engagement. Participants’ activity levels may have been influenced by their knowledge that their sensor data were being collected, and sensor data collected on study phones do not fully capture patients’ daily activities. Participants received buprenorphine for a minimum of 2 weeks prior to consent, and the findings may not apply to participants earlier in their course of treatment.

### Conclusions

Overall, this novel study demonstrates promise for the concurrent collection of multimodal data streams in support of digital phenotyping for this population and provides important insights for improving engagement. However, social media was a limiting factor. Studies that can leverage these digital data sources can be valuable in understanding the clinical needs of individuals with OUD and helping address the ongoing opioid crisis in the United States.

## Appendix

Multimedia Appendix 1 Percentage of days phone was carried at least 8 hours. Orange line=median; green triangle=mean.

Multimedia Appendix 2 Percentage of days watch was worn at least 18 hours. Orange line = median; Green triangle = mean.

Multimedia Appendix 3 Percent of days watch was worn to sleep. Orange line = median; Green triangle = mean.

Multimedia Appendix 4 Mean hours that phone was carried per day. Orange line = median; Green triangle = mean.

Multimedia Appendix 5 Means hours that watch was worn per day. Orange line = median; Green triangle = mean.

Multimedia Appendix 6 Mean hours of sleep recorded per day. Orange line = median; Green triangle = mean.

Multimedia Appendix 7 Mean posts per downloader (MPPD) at baseline and follow-up on Facebook, Instagram and Twitter, by gender, age, ethnicity, race.

## Abbreviations

EHR: electronic health record
EMA: ecological momentary assessment
KPNC: Kaiser Permanente Northern California
MPPD: mean number of posts per downloader
OUD: opioid use disorder
RR: response rate

## References

[1] Key substance use and mental health indicators in the United States: results from the 2020 National Survey on Drug Use and Health. Substance Abuse and Mental Health Services Administration.

[2] Ahmad FB, Rossen LM, Spencer MR, Warner M, Sutton P (2022). Provisional drug overdose death counts. Centers for Disease Control and Prevention.

[3] Ker S, Hsu J, Balani A, Mukherjee SS, Rush AJ, Khan M, Elchehabi S, Huffhines S, DeMoss D, Rentería Miguel E, Sarkar J (2021). Factors that affect patient attrition in buprenorphine treatment for opioid use disorder: a retrospective real-world study using electronic health records. Neuropsychiatr Dis Treat.

[4] Samples H, Williams AR, Olfson M, Crystal S (2018). Risk factors for discontinuation of buprenorphine treatment for opioid use disorders in a multi-state sample of Medicaid enrollees. J Subst Abuse Treat.

[5] Timko C, Schultz NR, Cucciare MA, Vittorio L, Garrison-Diehn C (2016). Retention in medication-assisted treatment for opiate dependence: A systematic review. J Addict Dis.

[6] Weinstein ZM, Kim HW, Cheng DM, Quinn E, Hui D, Labelle CT, Drainoni M, Bachman SS, Samet JH (2017). Long-term retention in Office Based Opioid Treatment with buprenorphine. J Subst Abuse Treat.

[7] Onnela J (2021). Opportunities and challenges in the collection and analysis of digital phenotyping data. Neuropsychopharmacology.

[8] Marsch LA (2018). Opportunities and needs in digital phenotyping. Neuropsychopharmacology.

[9] Marsch LA (2021). Digital health data-driven approaches to understand human behavior. Neuropsychopharmacol.

[10] Bertz JW, Epstein DH, Reamer D, Kowalczyk WJ, Phillips KA, Kennedy AP, Jobes ML, Ward G, Plitnick BA, Figueiro MG, Rea MS, Preston KL (2019). Sleep reductions associated with illicit opioid use and clinic-hour changes during opioid agonist treatment for opioid dependence: measurement by electronic diary and actigraphy. J Subst Abuse Treat.

[11] Jones A, Remmerswaal D, Verveer I, Robinson E, Franken IHA, Wen CKF, Field M (2019). Compliance with ecological momentary assessment protocols in substance users: a meta-analysis. Addiction.

[12] Shiffman S, Stone AA, Hufford MR (2008). Ecological momentary assessment. Annu Rev Clin Psychol.

[13] Bertz JW, Epstein DH, Preston KL (2018). Combining ecological momentary assessment with objective, ambulatory measures of behavior and physiology in substance-use research. Addict Behav.

[14] Kennedy AP, Epstein DH, Jobes ML, Agage D, Tyburski M, Phillips KA, Ali AA, Bari R, Hossain SM, Hovsepian K, Rahman MM, Ertin E, Kumar S, Preston KL (2015). Continuous in-the-field measurement of heart rate: correlates of drug use, craving, stress, and mood in polydrug users. Drug Alcohol Depend.

[15] Preston KL, Schroeder JR, Kowalczyk WJ, Phillips KA, Jobes ML, Dwyer M, Vahabzadeh M, Lin J, Mezghanni M, Epstein DH (2018). End-of-day reports of daily hassles and stress in men and women with opioid-use disorder: relationship to momentary reports of opioid and cocaine use and stress. Drug Alcohol Depend.

[16] Preston KL, Kowalczyk WJ, Phillips KA, Jobes ML, Vahabzadeh M, Lin J, Mezghanni M, Epstein DH (2017). Context and craving during stressful events in the daily lives of drug-dependent patients. Psychopharmacology (Berl).

[17] Epstein DH, Tyburski M, Craig IM, Phillips KA, Jobes ML, Vahabzadeh M, Mezghanni M, Lin J, Furr-Holden CDM, Preston KL (2014). Real-time tracking of neighborhood surroundings and mood in urban drug misusers: application of a new method to study behavior in its geographical context. Drug Alcohol Depend.

[18] Roth AM, Tran NK, Cocchiaro B, Mitchell AK, Schwartz DG, Hensel DJ, Ataiants J, Brenner J, Yahav I, Lankenau SE (2021). Wearable biosensors have the potential to monitor physiological changes associated with opioid overdose among people who use drugs: a proof-of-concept study in a real-world setting. Drug Alcohol Depend.

[19] Garg S, Taylor J, El Sherief M, Kasson E, Aledavood T, Riordan R, Kaiser N, Cavazos-Rehg P, De Choudhury M (2021). Detecting risk level in individuals misusing fentanyl utilizing posts from an online community on Reddit. Internet Interv.

[20] Reece AG, Reagan AJ, Lix KLM, Dodds PS, Danforth CM, Langer EJ (2017). Forecasting the onset and course of mental illness with Twitter data. Sci Rep.

[21] Hassanpour S, Tomita N, DeLise T, Crosier B, Marsch LA (2019). Identifying substance use risk based on deep neural networks and Instagram social media data. Neuropsychopharmacol.

[22] Ricard BJ, Marsch LA, Crosier B, Hassanpour S (2018). Exploring the utility of community-generated social media content for detecting depression: an analytical study on Instagram. J Med Internet Res.

[23] Thompson CM, Romo LK (2016). College students' drinking and posting about alcohol: forwarding a model of motivations, behaviors, and consequences. J Health Commun.

[24] Chary M, Genes N, Giraud-Carrier C, Hanson C, Nelson LS, Manini AF (2017). Epidemiology from tweets: estimating misuse of prescription opioids in the USA from social media. J Med Toxicol.

[25] Hsu M, Ahern DK, Suzuki J (2020). Digital phenotyping to enhance substance use treatment during the COVID-19 pandemic. JMIR Ment Health.

[26] Nelson BW, Low CA, Jacobson N, Areán P, Torous J, Allen NB (2020). Guidelines for wrist-worn consumer wearable assessment of heart rate in biobehavioral research. NPJ Digit Med.

[27] Marsch LA, Chen C, Adams SR, Asyyed A, Does MB, Hassanpour S, Hichborn E, Jackson-Morris M, Jacobson NC, Jones HK, Kotz D, Lambert-Harris CA, Li Z, McLeman B, Mishra V, Stanger C, Subramaniam G, Wu W, Campbell CI (2022). The feasibility and utility of harnessing digital health to understand clinical trajectories in medication treatment for opioid use disorder: d-tect study design and methodological considerations. Front Psychiatry.

[28] Gordon NP (2020). Similarity of adult Kaiser Permanente members to the adult population in Kaiser Permanente’s Northern California service area: comparisons based on the 2017/2018 cycle of the California health interview survey. Kaiser Permanente Division of Research.

[29] Jacobson NC, Bentley KH, Walton A, Wang SB, Fortgang RG, Millner AJ, Coombs G, Rodman AM, Coppersmith DDL (2020). Ethical dilemmas posed by mobile health and machine learning in psychiatry research. Bull World Health Organ.

[30] (2022). Health API features. Garmin Connect Developer Program.

[31] Raugh IM, James SH, Gonzalez CM, Chapman HC, Cohen AS, Kirkpatrick B, Strauss GP (2021). Digital phenotyping adherence, feasibility, and tolerability in outpatients with schizophrenia. J Psychiatr Res.

[32] Anderson Goodell EM, Nordeck C, Finan PH, Vandrey R, Dunn KE, Thrul J (2021). Feasibility and acceptability of using smartphone-based EMA to assess patterns of prescription opioid and medical cannabis use among individuals with chronic pain. Internet Interv.

[33] Roberts A, Rogers J, Mason R, Siriwardena AN, Hogue T, Whitley GA, Law GR (2021). Alcohol and other substance use during the COVID-19 pandemic: a systematic review. Drug Alcohol Depend.

[34] Sun S, Folarin AA, Ranjan Y, Rashid Z, Conde P, Stewart C, Cummins N, Matcham F, Dalla Costa G, Simblett S, Leocani L, Lamers F, Sørensen PS, Buron M, Zabalza A, Guerrero Pérez AI, Penninx BW, Siddi S, Haro JM, Myin-Germeys I, Rintala A, Wykes T, Narayan VA, Comi G, Hotopf M, Dobson RJ (2020). Using smartphones and wearable devices to monitor behavioral changes during COVID-19. J Med Internet Res.

