# Supplementary figures and images for: Patient Engagement in a Multimodal Digital Phenotyping Study of Opioid Use Disorder
Source: J Med Internet Res. 2023 Jun 13;25:e45556. doi: 10.2196/45556 (PMC10337375; doi:10.2196/45556)

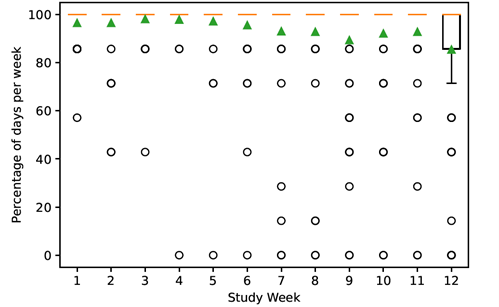

Supplement: Multimedia Appendix 1 [file jmir_v25i1e45556_app1.png]

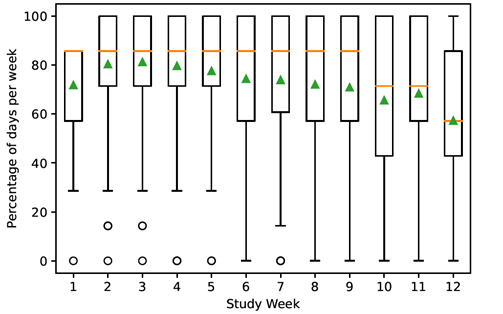

Supplement: Multimedia Appendix 2 [file jmir_v25i1e45556_app2.png]

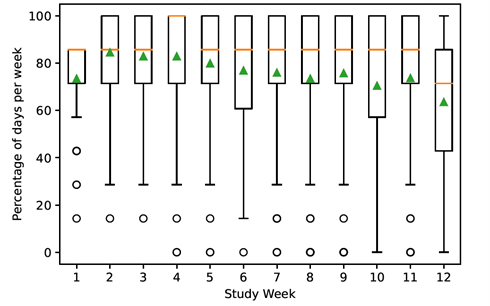

Supplement: Multimedia Appendix 3 [file jmir_v25i1e45556_app3.png]

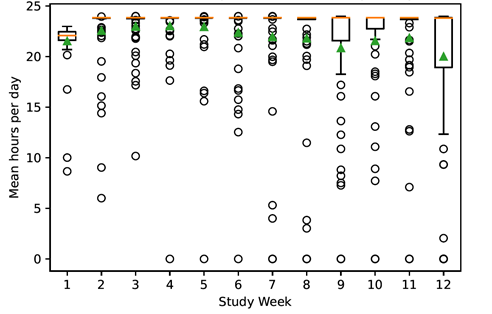

Supplement: Multimedia Appendix 4 [file jmir_v25i1e45556_app4.png]

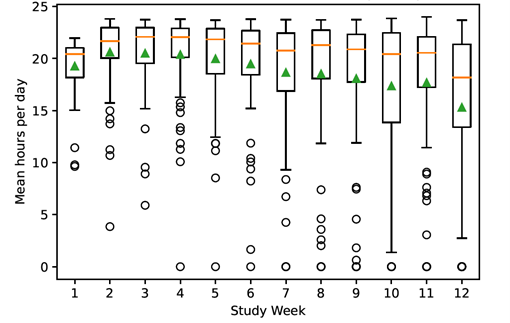

Supplement: Multimedia Appendix 5 [file jmir_v25i1e45556_app5.png]

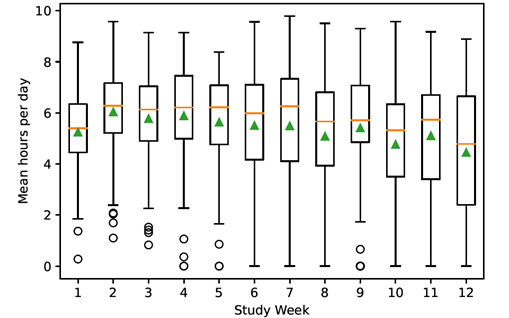

Supplement: Multimedia Appendix 6 [file jmir_v25i1e45556_app6.png]
