# Supplementary material for: Patient Engagement in a Multimodal Digital Phenotyping Study of Opioid Use Disorder
Source: J Med Internet Res. 2023 Jun 13;25:e45556. doi: 10.2196/45556 (PMC10337375; doi:10.2196/45556)
Supplement: Multimedia Appendix 7 [file jmir_v25i1e45556_app7.docx]

**Table A1. Mean Posts Per Downloader (MPPD)^a^ at Baseline and Follow-up** **on Facebook, Instagram and Twitter, by Gender, Age, Ethnicity, Race**

|  | **Facebook** | | | | | | **Instagram** | | | | | | **Twitter** | | | | | |
| --- | --- | --- | --- | --- | --- | --- | --- | --- | --- | --- | --- | --- | --- | --- | --- | --- | --- | --- |
|  | **Baseline** | | | **Follow-up** ^e^ | | | **Baseline** | | | **Follow-up** ^e^ | | | **Baseline** | | | **Follow-up** ^e^ | | |
|  | MPPD | (SD) | n**^b^** | MPPD | (SD) | n**^b^** | MPPD | (SD) | n**^b^** | MPPD | (SD) | n**^b^** | MPPD | (SD) | n**^b^** | MPPD | (SD) | n |
| **All downloaders** | 1263 | (1603) | 23 | 1218 | (1532) | 18 | 213 | (410) | 19 | 270 | (464) | 16 | 316 | (574) | 13 | 226 | (582) | 13 |
| **Gender Identity^c^** |  |  |  |  |  |  |  |  |  |  |  |  |  |  |  |  |  |  |
| Female | 1509 | (1854) | 14 | 1925 | (1895) | 9 | 176 | (260) | 11 | 236 | (293) | 8 | 361 | (545) | 6 | 416 | (766) | 7 |
| Male | 552 | (556) | 7 | 565 | (557) | 7 | 68 | (141) | 6 | 97 | (134) | 6 | 387 | (748) | 5 |  |  |  |
| **Age Group (years)** |  |  |  |  |  |  |  |  |  |  |  |  |  |  |  |  |  |  |
| 18-29 | 783 | (724) | 9 | 1015 | (740) | 5 | 150 | (270) | 11 | 281 | (332) | 6 | 674 | (711) | 6 | 574 | (878) | 5 |
| 30-49 | 1700 | (2065) | 12 | 1382 | (1905) | 11 | 335 | (595) | 7 | 312 | (608) | 8 | 10 | (16) | 6 | 10 | (14) | 7 |
| ≥50 | ^d^ |  |  | ^d^ |  |  | ^d^ |  |  | ^d^ |  |  | ^d^ |  |  | ^d^ |  |  |
| **Ethnicity** |  |  |  |  |  |  |  |  |  |  |  |  |  |  |  |  |  |  |
| Hispanic, Latino or of Spanish Origin | ^d^ |  |  | 680 | (262) | 5 | ^d^ |  |  | 584 | (715) | 5 | ^d^ |  |  | ^d^ |  |  |
| Not Hispanic, Latino or Spanish Origin | 1373 | (1748) | 19 | 1425 | (1770) | 13 | 93 | (186) | 15 | 127 | (217) | 11 | 410 | (630) | 10 | 325 | (688) | 9 |
| **Race** |  |  |  |  |  |  |  |  |  |  |  |  |  |  |  |  |  |  |
| White | 1336 | (1712) | 17 | 1482 | (1696) | 13 | 279 | (464) | 14 | 360 | (542) | 11 | 225 | (563) | 9 | 13 | (18) | 8 |
| Non-white/More than one race | 1056 | (1361) | 6 | 531 | (727) | 5 | 30 | (27) | 5 | 73 | (56) | 5 | ^d^ |  |  | 568 | (883) | 5 |

**^a^** Mean number of posts (total number of posts in a given subgroup, divided by the number of downloaders in the subgroup) since the account was established.

**^b^** n represents the number of people who downloaded each type of social media data at each time point.

**^c^** Values are not reported for participants who reported their gender identity as “Non-Binary” or “Prefer not to report” due to small cell sizes.

^d^ Not reported due to small cell sizes.

^e^ MPPD could be higher at follow up than baseline, because different people may have downloaded at each timepoint.
